# microRNAs in Neurodegeneration: Current Findings and Potential Impacts

**DOI:** 10.4172/2161-0460.1000420

**Published:** 2018-01-23

**Authors:** Salil Sharma, Hui-Chen Lu

**Affiliations:** Department of Psychological and Brain Sciences, The Linda and Jack Gill Center for Bimolecular Sciences, Indiana University, Bloomington, IN 47405, USA

**Keywords:** microRNAs, MiRs, Dementia, Neurodegeneration

## Abstract

Significant advancements have been made in unraveling and understanding the non-coding elements of the human genome. New insights into the structure and function of noncoding RNAs have emerged. Their relevance in the context of both physiological cellular homeostasis and human diseases is getting appreciated. As a result, exploration of noncoding RNAs, in particular microRNAs (miRs), as therapeutic agents or targets of therapeutic strategies is under way. This review summarizes and discusses in depth the current literature on the role of miRs in neurodegenerative diseases.

## Introduction

The sequencing of the human genome has led to the swift discovery and unprecedented research into many protein-coding genes. However, protein-coding genes account for only 2% of the entire genome. The untranslated genome, that was once unappreciated and thought to be of limited importance, has gained crucial functional relevance in recent years [1,2]. The non-protein coding genome comprises diverse sets of non-coding structures that include miRs, transcribed ultraconserved regions (T-UCRs), small nucleolar RNAs (snoRNAs), P-element induced wimpy testes (PIWI)-interacting RNAs (piRNAs), large intergenic non-coding RNAs (lincRNAs) and long non-coding RNAs (lncRNAs) [3]. Among these, the most widely studied group of noncoding RNAs are the miRs. These molecules have been shown to regulate a variety of physiological functions including development, growth, metabolism and cellular homeostasis [4,5]. In addition, their dysregulated processing and altered abundance has become a distinguishing characteristic of many diseases, such as cancer, cardiovascular and neurological disorders [6–8]. The roles of miRs are nascent and understudied in neurodegenerative diseases. Recent studies have begun to highlight their regulatory functions and impact on neurodegenerative diseases. In this review, we will focus on the emerging roles of miRs in neurodegenerative diseases with an emphasis on their regulatory function, and their potentials as biomarkers in disease progression.

## miR Biogenesis

**Figure 1:**
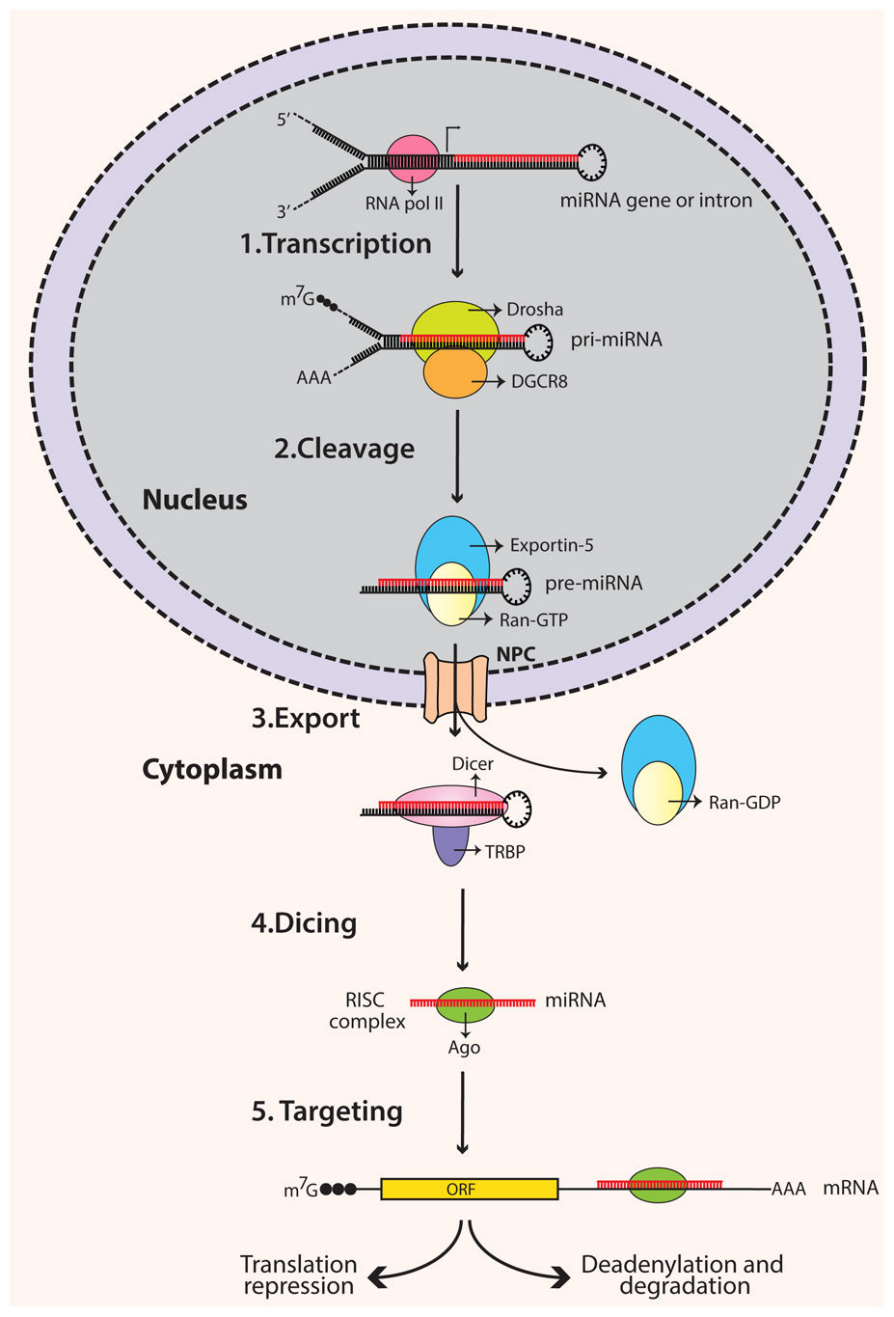
microRNA biogenesis pathway. MiR genes are transcribed in the nucleus by RNA polymerase II enzyme. The resulting pri-miR is enzymatically cleaved by the enzyme Drosha in conjunction with its partner DGCR8 to produce ~65 nucleotide long pre-miR. Pre-miR is exported into the cytoplasm via nuclear pores with the help of nuclear transport receptor Exportin 5. In the cytoplasm, RNase III Dicer along with accessory protein TRBP further slices pre-miR into mature miR. Mature miR is loaded into RNA induced silencing complex (RISC) for initiating target gene downregulation. The main protein of RISC complex, Argaunote, guides the mRNA to the RISC complex and mediates mRNA degradation or translation repression by miR

miRs are endogenous, small noncoding RNA molecules ranging between 18–23 nucleotides [9,10]. They regulate gene expression by repressing the translation of their complementary target genes [11]. Precursors of miRs have characteristic stem-loop structures (Figure 1). Precursor miRs are generated in the nucleus by multiple processing steps and are then exported into the cytoplasm where they are further processed to become mature miRs [12]. Based on the genomic location, miRs can either be intergenic or located in transcriptional units (TUs) [9,13]. The majority of miRs are located in defined TUs, and can be further classified as: (1) Intronic miRs in coding TU, (2) Intronic miRs in non-coding TU, (3) Exonic miRs in coding TU, and (4) Exonic miRs in non-coding TU [14]. As mentioned, miR processing takes place in the nucleus, where RNA polymerase II (pol II) transcribes miR genes into primary miRs (pri-miRs). Pol II transcribed pri-miRs are 5′ capped and 3′ polyadenylated [15]. Pri-miR is cleaved by RNase-III enzyme Drosha to an approximately 60–70 nucleotide structure called pre-miR. Drosha is guided by its cofactor protein called DiGeorge syndrome critical region in gene 8 (DGCR8) to the base of the stem-loop structure of pri-miR for cleavage [16–18]. The formation of pre-miR initiates its transport from nucleus to cytoplasm. Large protein channels called nuclear pore complexes, embedded in the nuclear membrane, serve as channels for pre-miR export. The export is mediated by nuclear transport receptor exportin-5 (EXP5) and its cofactor Ran (GTP-bound form) with the energy provided by GTP hydrolysis [19,20]. In the cytoplasm, pre-miRs are further processed by endonuclease cytoplasmic RNase III enzyme Dicer to form mature miRs. Dicer is a highly-conserved enzyme in eukaryotic organisms and works in conjunction with various proteins [21,22]. In humans, Dicer partners with trans-activation response RNA-binding protein (TRBP) for its processing activity [23,24]. Cleavage by Dicer leads to the formation of approximately 22-nucleotide miR duplex [25,26]. Next, the miR-duplex is loaded into the RNA induced silencing complex (RISC). RISC is a multi-protein effector complex with Argonaute (Ago) family proteins as the major constituents [27]. In the majority of cases, in the RISC complex the passenger strand of the miR-duplex is degraded, whereas the guide strand remains bound to Ago to form the functional mature miR. The unwinding of the miR-duplex and degradation of the passenger strand is mediated by putative RNA helicases, such as helicase A (RHA) [28]. The incorporation of the mature miR strand into the RISC complex prepares the complex for target mRNA recognition.

Gene silencing by miRs takes place in the RISC effector complex. The two most widely studied mechanisms of target gene repression are: (1) mRNA degradation, and (2) translation repression. Both mechanisms lead to down-regulation of target genes. The pairing of miR and mRNA can induce endolytic cleavage of the messenger transcripts, and is referred to as ‘slicer activity’ [29]. The cleaved mRNA undergoes deadenylation followed by degradation by exonucleases [30]. The translational repression of the target mRNA can take place at various stages of translation that include initiation, elongation and termination [31]. Alternatively, translation repression can also occur by sequestering of miR-mRNA protein complexes into processing bodies called P-bodies [29,32,33]. In this situation, the spatial separation of mRNA from translational machinery prevents its translation.

## Neurodegeneration

Around 70% of annotated miRs have been detected in the brain [34]. Here, they play a dynamic role in neuroplasticity, neurodevelopment, synaptic development, including, synapse formation and synapse maturation, dendrite spine formation, and stress responses [35,36]. Altered miR levels have been observed in a variety of neurological disorders [37–40]. With advanced parallel sequencing techniques, a cluster of human-brain specific miRs has been detected, highlighting the evolving complexity of the roles of miRs in neuronal functions [41]. Interestingly, a very small percentage of miRs are enriched in brain, suggesting that miR mediated transcriptional control of many target genes and brain-specific regulatory pathways are under tight control [42–44]. In addition to highly conserved miRs, emerging non-conserved miRs are being rapidly discovered [45,46]. The subset of brain-expressed and -enriched miRs points to their specific roles in brain development and function [47,48]. Many of them show cell-type [49] or brain-region specificities [50]. The majority of neuronal miRs are distributed in a gradient through the somatodendritic compartment with high levels in soma and low levels in distal dendrites [51]. However, a very small number of miRs are enriched in the dendrites.

Several mammalian studies have dissected the mechanistic role(s) of miRs in neuroprotection and neurodegeneration. It was shown in animal models that endoribonuclease Dicer, a key RNase to produce mature miRs is essential for the maintenance of forebrain excitatory neuron survival in the adult cortex. Its loss results in impaired neurogenesis and massive neuronal loss [52]. In another study, loss of Dicer in the adult brain led to neurodegenerative phenotype, displayed by neuronal loss in hippocampus, cell shrinkage in cortex and hyperphosphorylation of tau protein associated with neurofibrillary pathology [39]. In the following sections, we will summarize studies elucidating the potential roles of specific miRs in neurodegeneration in various animal models and human diseases (Table 1).

## Alzheimer’s Disease (AD)

AD is a progressive neurodegenerative disease with multiple factors associated with its development and pathogenesis [53]. At the cellular level, a progressive and substantial loss of cortical neurons occurs in AD [54,55]. Maximal degeneration occurs in the cortex and hippocampus, leading to memory and learning deficits [56,57]. Most studies have theorized the deposition of proteins, including tau and amyloid within and outside the neurons as presymptomatic changes. These changes subsequently progress to full clinical manifestation of AD [58]. Many neuroimaging and biomarker techniques are being used and designed for the early diagnosis of AD. These include Magnetic Resonance Imaging, Positron Emission Tomography, and cerebrospinal fluid (CSF) and blood-based protein biomarkers [59]. Several studies have looked into the altered miR levels in the brains and body fluids of AD patients as well as the regulatory role of miRs in various aspects of AD pathogenesis, including, inflammation, lipid metabolism, oxidative stress, and protein dysregulation. Through these recent studies, many miRs have emerged as strong candidates that target individual genes or gene-network pathways involved in the cause and progression of neurodegenerative diseases. For example, in cases of sporadic AD, the miR-29a/b-1 cluster was significantly decreased, correlating with high levels of the enzyme β-secretase 1 (BACE1) and the production of β-amyloid peptide associated with disease pathogenesis [60,61]. Comparison of miR abundance in fetal, adult and AD hippocampi showed that miR-9, miR-124a, miR-125b, miR-128, miR-132 and miR-219 are altered, and may contribute to neuronal dysfunction [62].

AD brains have been shown to contain low levels of brain-derived neurotrophic factor (BDNF), an important protein involved in stimulating neurogenesis and modulating cognitive function [63–65]. Lee et al. showed that BDNF mRNA is post-transcriptionally targeted by miR-206 and BDNF reduction by miR-206 may result in AD-like progression in mice. Conversely, enhancing BDNF levels by miR-206 suppression via miR-206 specific antagomirs increased synaptic density, enhanced hippocampal neurogenesis, and improved memory function in a mouse model of AD [66]. Reactive oxygen species (ROS) could result in upregulation of miR-9, miR-125b and miR-128 in cultured neurons and suggests the possibility that miRs may mediate ROS’s pathogenic effects in AD [67].

Tau is an important protein involved in tauopathies and neurodegenerative disorders [68,69]. Santa-Maria et al. demonstrated tau as a bona fide target gene of miR-219, a highly conserved miR in many species. MiR-219 was shown to be downregulated in the autopsy brains of AD patients. In a Drosophila model of tau overexpression, reducing miR-219 levels led to significant increases in tau transcripts and aggravation of tau toxicity. Conversely, overexpression of miR-219 in this model remarkably ameliorated toxic effects caused by tau overexpression [70]. Thus, inhibition of tau expression by miR approach could be a step toward therapeutic intervention in tauopathies. Lau et al. have shown deregulation of 35 miRs in the hippocampus, prefrontal cortex and temporal gyrus in a cohort of 41 late-onsets AD (LOAD) patients compared to 23 controls [71]. In addition, they found 41 miRs altered in an independent cohort of 49 patients categorized by six Braak stages (BRI to BRVI). MiR132-3p was strongly deregulated in both cohorts, and its altered expression was mainly observed in neurons exhibiting hyper-phosphorylation of tau protein. Many miR-132 gene targets, based on miR target prediction tools, are relevant to AD [71]. These include MAPT (gene coding for tau protein) and Tau post-transcriptional regulators, including EP300, SIRT1 and GSK3B and various members of the Forkhead (Fox) transcription factor (TF) family. Similar findings were also presented by Wong et al. where miR-132 and miR-212 were downregulated in cortical areas and CA1 hippocampal neurons of human AD brains [72]. Primary neuronal culture studies identified many targets of miR-132 and miR-212 that are involved in Akt survival signaling pathway, including PTEN, FOXO3A and EP300. Silencing of these proteins in neurons was sufficient to suppress apoptosis caused by miR-132/212 downregulation [72]. Tau was identified as a bona fide target of miR-132. Deficiency of miR-132 and miR-212 in mice resulted in an increase in the expression, phosphorylation and pathological aggregation of tau. Conversely, miR-132 administration in a murine model of AD improved long term memory deficits; highlighting the emerging role of miR-132/212 family in tau related neurodegenerative diseases [73]. The downregulation of miR-132 is widely observed in many separate studies either in human brains displaying neurodegeneration or in animal models of neurodegeneration. Genome wide studies on the miR signature in the prefrontal cortex of AD and control brains also identified significant downregulation of miR-132 and miR-212 in AD brains [74]. Salta et al. showed that miR-132 loss leads to upregulation of its target, inositol 1,4,5-trisphosphate 3-kinase B (ITPKB) in an AD mouse model. ITPKB is a regulator of BACE1 activity and tau phosphorylation. Its induction via miR-132 loss intensifies both amyloid burden and tau pathology [75].

AD also shares many pathological characteristics with age-related macular degeneration (AMD), a neurodegenerative disorder of visual system. These include: presence of amyloid β (Aβ) in senile plaques in AD brains and in the drusen in age-related macular degeneration (AMD), elevation of cholesterol and other oxidized lipid metabolites and aggravated inflammatory signalling [76]. miR-9, miR-125b, miR-146a and miR-155 are abundant in human brain and retina, and their progressive induction has been implicated in both AD and AMD. Interestingly, miR-146a and miR-155 supports inflammatory neurodegeneration via targeting complement factor H (CFH), a major negative regulator of the innate immune and inflammatory response [77].

Triggering Receptor Expressed on Myeloid Cells 2 protein (TREM2) plays an important role in immune surveillance and phagocytic functions of microglia cells in the brain and has been recognized for its importance in neuroinflammation [78,79]. Many studies have shown an association of TREM2 with the neuroinflammatory axis of neurodegeneration, including AD [80,81]. TREM2 contains binding site for miR-34a mature sequence, and was experimentally shown to be a direct target of miR-34a. miR-34a can be regulated by a proinflammatory transcription factor, nuclear factor-kB (NF-KB) and its level was significantly upregulated in AD hippocampi compared with aged matched controls [82]. Thus, miR-34a mediated TREM2 downregulation could possibly lead to impaired phagocytic and inflammatory response in the progression of AD [83]. Zovoilis et al. conducted an in-depth quantification of the mouse hippocampus miRNAome and identified miR-34c as the miR highly linked to hippocampal function with several predicted targets belonging to hippocampal memory formation [84]. MiR-34c upregulation was observed in the hippocampus of a mouse AD model and human AD patients. Suppressing miR-34c levels in mouse AD model led to an improvement of cognitive impairment and improvement in associated learning. This could, in part, be due to reestablishment of physiological levels of SIRT1 with miR-34c inhibition [84]. SIRT1 is a bona fide target of miR-34c and it is essential for normal cognitive function and synaptic plasticity [85]. It was shown that a group of six proinflammatory miRs are upregulated in the brain of sporadic AD brains compared to the control brains (miR-7, miR-9, miR-34a, miR-125b, miR-146a and miR-155) [86,87]. The downregulated targets of these miRs can have global anomaly in gene expressions that could then culminate in the widespread neuropathological implications observed in AD.

With respect to inflammatory dysfunction, complement factor H (CFH) has been shown to be downregulated in both degenerating AD brains and retina [88,89]. CFH belongs to a group of proteins that form a part of the regulator of complement activation (RCA). Decreased levels of CFH result in activation of complement proteins that are a part of the innate immunity system, causing an elevated inflammatory response. A subset of four miRs namely: miR-9, miR-125b, miR-146a and miR-155 have been shown to directly or indirectly repress CFH expression, thus driving inflammatory responses associated with AD and AMD [77]. Lehmann et al. showed elevated levels of miR let-7 levels in the CSF of AD patients [90]. They also reported that let7 is capable of activating intrinsic cell death pathway in cultured neurons via Toll-like receptor 7 (TLR7). Such stimulation results in increased production of inflammatory cytokines by microglia and increased cell death in hippocampal neuronal culture. *In vivo*, intrathecal application of let7 led to marked neuronal loss and axonal injury in cerebral cortex of mice through activation of its unconventional target TLR7 [81,82,90,91]. Thus, miRs can also contribute to neurodegeneration via receptor targets that are activated independent of post-transcriptional regulation by miRs.

Dysregulation of Aβ peptide metabolism plays an immense role in AD pathogenesis. Apolipoprotein E (ApoE) lipidation constitutes an important role in brain lipid metabolism, and critically influence Aβ metabolism. With genetic deletion and pharmacological inhibition of miR-33 in mice, Kim et al. [92] showed that miR-33 antagonism can effectively increase ApoE lipidation and reduce Aβ levels by upregulation of its target, ATP-binding cassette transporter A1 (ABCA1) in the brain. ABCA1 is a membrane-associated lipid pump that maintains cholesterol homeostasis by mediating efflux of lipids from cells to apolipoprotein A1 (APOA1) and APOE. By regulating ABCA1 expression, miR-33 plays an important role in lipid metabolism associated with AD pathogenesis. Disrupted cholesterol homeostasis can lead to inefficient neurotransmission, attenuated synaptic plasticity and also results in neurodegenerative conditions including AD, Huntington’s disease (HD) and Parkinson’s disease (PD) [93–96]. For example, the polymorphic alleles of the enzyme APOE, involved in the transport of cholesterol, are strong risk factors in AD [97,98]. *In vitro* studies on neuronal cells have shown that ABCA1 gene is a bona fide target of miR-106b. Inhibition of ABCA1 gene by miR106b overexpression led to an impairment of cellular cholesterol efflux and Aβ clearance [99]. Ramirez et al. [100] demonstrated that miR-758 regulates the post-transcriptional expression of ABCA1 by directly targeting its 3′ UTR. Moreover, miR-758 regulates cellular cholesterol efflux in neural cells and is involved in neuronal cholesterol homeostasis. Thus, a few miRs participate in the regulation of cholesterol homeostasis in the brain, and their altered expression may contribute to neurodegeneration.

## Parkinson’s Disease

Parkinson ’s disease (PD) is a neurological disorder characterized by extensive loss of dopaminergic neurons in the substantia nigra pars compacta midbrain region, resulting in severe motor symptoms [101,102]. The neurons in the substantia nigra region display the presence of intracytoplasmic inclusions known as Lewy bodies [101]. Mutations in the gene that encodes α-synuclein, a synaptic protein, and subsequent misfolding of this protein are involved in autosomal-dominant form of Parkinson’s disease [103,104]. In addition, many other insults, including oxidative stress, environmental toxins and mitochondrial dysfunction have been implicated in this neurodegenerative disorder [101].

Recent studies have focused on the role of miRs in PD pathogenesis. MiR-133b is specifically expressed in midbrain dopaminergic neurons. However, midbrains from PD patients were shown to be deficient in miR-133b expression. *In vivo* miR-133b deletion resulted in reduction of tyrosine hydroxylase and dopamine transporter levels, implicating its discreet role in dopaminergic function and neurodegeneration [105]. Several neurotoxins are believed to induce the oxidative stress that plays an important role in PD progression [106]. Oxidative stress induced by neurotoxins can contribute significantly to PD pathogenesis. Among many genes implicated in PD, DJ-1 is believed to be an important molecular chaperone and oxidative sensor that has a protective response to oxidative stress. DJ-1 is implicated in both familial and sporadic PD [107]. Multiple studies have linked the altered functions of DJ-1 to the development of PD [107,108]. Loss of function mutations in DJ-1 as well as its lower expression in the substantia nigra of sporadic PD patients, may contribute to the disease pathogenesis [109]. Xiong et al. showed that miR-494, which is abundantly present in substantia nigra pars compacta (SNpc), targets DJ-1. Gain of miR-494 function led to decreased DJ-1 protein levels and caused increased oxidative stress as well as loss of dopaminergic (DA) neurons in both *in vitro* and *in vivo* studies, thus confirming miR-mediated regulation of DJ-1 in PD pathogenesis [110].

As mentioned before, PD is characterized by excessive loss of DA neurons in substantia nigra compacta and accumulation of α-synuclein in Lewy bodies and neuritis. This leads to excessive neuroinflammation that contributes to PD pathogenesis [111]. In vitro studies have shown that α-synuclein can induce interleukin-1β production by macrophages, which in part is dependent on inflammasomes containing nod-like receptor protein 3 (NLRP3) [112]. With gain and loss of function *in vitro* studies it was demonstrated that NLRP3 is a target of miR-7. In a mouse PD model, miR-7 overexpression attenuated dopaminergic neuron degeneration, highlighting the role of this miR in mediating NLRP3 dependent inflammation in PD [113]. In a small cohort, Cho et al. [114] have shown the downregulation of miR-205 in the frontal cortex of PD patients when compared to controls. At the same time, protein levels of Leucine-rich repeat kinase 2 (LRRK2) genes, which is implicated in alpha-synuclein-mediated neurodegeneration and sporadic PD [115,116], are elevated. The 3′ UTR of LRRK2 gene has a conserved binding site for miR-205 across many vertebrate species. *In vitro* studies confirmed the repression of LRRK2 protein levels by miR-205. In addition, miR-205 treatment restores neurite outgrowth deficits caused by LRRK2 mutant over-expression. Gehrke et al. [40] showed that interaction of gain-in-function mutation forms of LRRK2 with various miRs could regulate protein synthesis of downstream miR targets, leading to pathogenic effects of LRRK2. In Drosophila, cell cycle and survival control proteins, E2F1 and DP are translationally repressed by let-7 and miR-184*. Antagonistic interaction of pathogenic LRRK2 with let-7 and miR-184* could lead to overproduction of E2F1 and DP, resulting in degeneration of dopaminergic neurons [40]. Thus, LRRK2 could employ miR-regulated protein machinery for its pathogenic effects in PD.

In search of miR biomarkers for PD, Margis et al. [117] evaluated blood samples from control, non-treated, early-onset and treated PD subjects. They concluded that the expression profiles of miR-1, miR-22* and miR-29 could be used to distinguish between non-treated PD patients from healthy subjects. Furthermore, they identified that miR-16-2*, miR-26a2* and miR-30a expression levels could differentiate treated from untreated PD patients [117].

## Amyotrophic Lateral Sclerosis (ALS)

ALS is a neurodegenerative disease characterized by dysfunction of human motor neurons in the brain and spinal cord [118]. In ALS, there is a failure of the proteasome system to recycle defected proteins, manifested by the presence of ubiquitinated inclusions (UBIs). Mutations of many RNA-binding proteins may play causative roles in ALS. These include mutations in TAR DNA binding protein (TDP-43), superoxide dismutase 1(SOD1), heterogeneous nuclear ribonucleoprotein A1 (hnRNPA1) and fused in sarcoma (FUS) genes [119–123]. In addition, hexanucleotide expansion on chromosome 9 in open reading frame 72 (C9ORF72) also accounts for familial cases of ALS [124,125]. In response to cellular stress, stress granules are formed, which are involved in the modulation of mRNA translation [126,127]. Many mutated RNA-binding proteins that are implicated in ALS are recruited to stress granules [122,128]. These stress granules are observed in pathological ALS samples, and are involved in the pathogenesis of disease [128–130]. Many proteins that constitute RNA induced silencing (RNAi) machinery are involved in stress signaling cascades [131]. miR down-regulation was consistently found in both familial and sporadic cases of ALS. Various stress conditions were shown to alter the localization and dynamics of the miR processing enzyme DICER and AGO2 protein [132,133] as well as expression of mature functional miRs. Many of the ALS causing mutated proteins had similar effects on DICER activity and miR biogenesis. The interactions between these proteins and DICER in the stress granules are likely to contribute to the reduced DICER activity, and global miR downregulation in ALS [134].

TDP-43 is a ubiquitously expressed protein that is involved in RNA-binding, and therefore, regulates mRNA transcription and alternative splicing. TDP-43 has been found to be associated with complexes containing miR-processing ribonucleases, Drosha and Dicer and hence, takes part in miR biogenesis. *In vitro* depletion of TDP-43 results in the dysregulation of many miRs. Freischmidt et al. [135] confirmed the dysregulation of many TDP-43 regulated miRs from *in vitro* studies in samples of CSF and serum obtained from ALS patients. These include, miR-132-5p, miR-132-3p, miR-143-5p, miR-143-3p in CSF and serum and miR-574-5p in CSF. Thus, these miRs could potentially be the biomarkers of decreased TDP-43 function in ALS patients.

Williams et al. [136] investigated changes in miRs in a mouse model of ALS with low copy overexpression of mutant form of SOD1. This model recapitulates the progression of human ALS symptoms. Out of the 320 differentially expressed miRs, miR-206 was further investigated by virtue of its most dramatic upregulation. Loss of miR-206 in a mouse ALS model induced the expression of its target, histone deacetylase 4 (HDAC4) and accelerated skeletal muscle atrophy and disease progression. miR-206 is required for efficient regeneration of neuromuscular synapses after acute nerve injury. Thus, miR-206 upregulation in ALS condition may slow ALS progression by promoting the compensatory regeneration of neuromuscular synapses.

Inhibition of miR biogenesis has been reported to cause spinal muscular atrophy, myofiber atrophy with signs of denervation, and spinal motor neuron (SMN) degeneration in SMN diseases including ALS [137]. Emde et al. [134] demonstrated that various forms of familial and sporadic human ALS are characterized by reduction of miR levels in motor neurons. Both chemical stressors and ectopic expression of ALS causing mutant genes were sufficient to cause reduction of these miRs and impaired miR biogenesis. Furthermore, enhancing Dicer-complex activity with enoxacin, an antibiotic known to increase miR biogenesis, rescued the impaired miR processing as a result of ALS causing mutant protein [134]. Therefore, inhibition of miR processing at the level of Dicer activity appears to be a common denominator in various forms of ALS.

## Huntington’s Disease (HD)

In HD, there is a progressive dysfunction and degeneration of basal ganglia. The gene for Huntington protein undergoes CAG expansion that results in an expanded polyglutamine tract in the encoded Huntington protein [138]. The pathological effects of this gene mutation are observed in many regions of the brain, but are most prominent in the striatum [139,140]. Widespread transcriptomic changes are observed in the brains of HD patients and mouse models [141–143]. Several of these changes are possibly regulated by miRs either directly or indirectly. REST is an important transcriptional repressor that silences the expression of neuronal genes in non-neuronal cells [144]. Wild type Huntington sequesters the transcriptional repressor REST (Repressor Element 1 Silencing Transcription Factor and is also called NRSF) in the cytosol. However, mutated Huntington genes are incapable of interacting with REST and thus, REST translocate into the nucleus. This leads to an abnormal decrease of BDNF transcription, a REST target gene and reduced striatal neuron survival [145]. In addition to BDNF, REST also represses the expression of many genes involved in neuronal function and survival [146]. In addition, REST also regulates the expression of many global and brain-restricted miRs including mir-132, mir-124a and mir-9 [147–149]. Conaco et al. [150] have shown the presence of functional REST-binding sites on multiple miRs, and have further confirmed the regulation of miR124a by REST. These studies highlight the impact of misregulation of REST localization on multiple mRNAs and miRs, leading to subsequent deleterious transcriptional alterations. A comprehensive study by Johnson et al. [147] has revealed that REST regulates a number of brain-restricted precursor-miRs *in vitro*. Furthermore, the expression of REST-regulated miRs is dysregulated in a mouse model of HD and in post-mortem tissue from HD patients. Through this approach, miR-132 was identified as a REST-regulated miR. The expression of miR-132 is repressed in the cortex of HD patients, accompanied with an increase in miR-132 target p250GAP [147]. p250GAP, a member of the Rac/Rho family of GAPs, is involved in the inhibition of neuronal outgrowth and may have implications in HD [151]. Packer et al. [152] performed a similar study in control and HD grade 1-4 Brodmann’s area 4 (BA4) cortex brain samples to assess the REST-regulated mature miRs. Many miRs were identified with differential expression at various stages of HD when compared to controls. MiR-9 and miR-9*, which are processed from the same primary transcript, were downregulated in early stages of HD. Both miRs have upstream RE1 sequences that can lead to transcriptional repression when occupied by REST. Interestingly, REST and CoREST (part of REST repressor complex) are also the targets of MiR-9 and miR-9* respectively. Using an *in vitro* system, the authors demonstrated that miR-9/miR-9* and Rest/CoRest undergo a negative feedback loop to balance each other’s expression levels, which may become altered in HD [152].

Gaughwin et al. [153] conducted an *in vitro* miR screen in a cell line over-expressing mutant Huntington gene. The differentially expressed miRs were evaluated for their presence, bio-stability and expression changes in human plasma samples from control and HD patients at different disease stages. Plasma miR-34b was found to be significantly elevated in HD patients at pre-manifest stage and stages II/III. *In vitro* experiments were used to show that inhibition of miR-34b abrogates the toxic effects of mutant Huntington overexpression. In an independent study, Hoss et al. [154] found differentially expressed 75 miRs in HD brains. Several miRs found in this study have been shown in HD by other studies, including miR-132-3p [147,155], miR-148a-5p [156], miR-150-5p [157], miR-214-5p [157] and miR-196a [158,159]. MiR-10b-5p, the highest upregulated miR found by Hoss et al. [160] has been shown to target BDNF and its enhanced cell survival of HD cell-line models. Future studies into the function of miR-10b may elucidate its role in HD pathogenesis.

## Prion Disease

Prion disease is characterized by the conversion of the normal cellular prion protein PrPC to the infectious Scrapie prion protein PrPSc. Many pathological features are observed in the central nervous system during the progression of Prion disease. These include deposition of a protease resistant form of the prion protein PrPRes, extensive microglia and astrocyte hypertrophy and vacuolation of neurons [161–163]. Synapse and neuronal loss have also been reported in the hippocampus of scrapie-infected murine models of Prion disease [164].

Majer et al. [165] performed a high throughput screen of the transcriptional changes of mRNA and miR levels that occur in the CA1 hippocampal region prior to the onset of Prion disease in a mouse model. Over the entire course of the disease, 88 differentially expressed miRs were identified. 17 miRs were also altered in the presymptomatic stage of Prion disease. The upregulation of miR-16-5p, miR-26a-5p, miR-29a-3p, miR-132-3p, miR-140-5p, miR-124a-3p and miR-146a-5p were validated. Examination of the Gene Ontology of their mRNA targets identified many pathways that were associated with neuronal function, including synaptic organization. In a recent independent study, Boese et al. [166] found that the abundance of miR-124a-3p, miR-136-5p and miR-376a-3p was elevated in the preclinical stage of Prion disease in a murine model [166]. At later stages of the disease, the levels of miR-146a-5p, miR-142-3p, miR-143-3p, miR-145a-5p, miR-451a, miR-let-7b, miR-320 and miR-150-5p were all increased. These miRs target genes that are involved in maintaining synaptic structural plasticity and dendritic spine densities in brain [166].

## Multiple System Atrophy (MSA) and Dentatorubral-pallidoluysian Atrophy (DRPLA)

MSA is a progressive neurodegenerative disorder characterized by substantial neural loss in many brain regions. There is oligodendrocytic accumulation of alpha-synuclein that results in autonomic dysfunction and motor abnormalities [167,168]. DRPLA is a neurological disorder that leads to progressive myoclonic epilepsy and spinocerebellar degeneration. It is an autosomal dominant disease that is caused by an expansion of polyglutamine stretch [169,170]. Widespread dysregulation of miRs was observed in MSA [171]. Expression levels of miR-96 were upregulated in MSA with a concomitant decrease in its targets genes, the solute carrier protein family members SLC1A1 and SLC6A6, in both MSA patients and a transgenic mouse model of MSA. Polymorphism in SLC1A1 has been linked to MSA, and SLC6A6 may have neuroprotective activity [171]. For DRPLA, many studies have employed simple model organisms like Drosophila to study the effect of miRs on gene targets associated with neurodegeneration. Atrophin-1 is implicated in the neurodegeneration pathogenesis associated with DRPLA [172]. miR-8 expressing cells reduce the levels of its direct functional target atrophin, a transcriptional co-repressor. Loss of miR-8 elevates the levels of atrophin, leading to extensive transcriptional changes, and causing apoptosis in the brain and many behavioral defects [173].

## Conclusion

Substantial progress has been made in the field of miR research, especially in the understanding of basic biology, function and disease relevance. Many recent technological advances have resulted in studying the miR gain and loss of function studies [174,175], epigenetic regulation [176], miR regulated intricate network pathways using systems biology and bioinformatics approaches [177–179], and development of various transcriptomic screens [180,181]. From the perspective of neurodegeneration, the mechanistic insight and impact of miRs remains to be determined. For developing miR-based biomarkers for neurodegenerative diseases in humans, higher powered miR-profiling screens are needed to assess miR-changes with greater confidence [182]. On-going research progress in studying non-coding RNA in brain and neurological disorders will lead to better understanding, diagnosis and treatment of neurodegenerative disorders.

## Figures and Tables

**Table 1:** microRNAs in neurodegeneration.

microRNA	Pathology	Direct/indirect targets	References
miR-206	AD	BDNF	[66]
miR-219	AD	MAPT	[70]
miR-132-3p, -212	AD	MAPT, EP300, SIRT1, GSK3B, FOX-TF, ITPKB	[71–75]
miR-9, -125b, -146a, -155	AD, AMD	CFH	[76,77]
miR-34a	AD	TREM2	[83]
miR-34c	AD	SIRT1	[85]
miR let-7	AD	TLR7	[90,91]
miR-33, -106b, -758	AD	ABCA1	[92,99,100]
miR-7, -9, -34a, -125b, -146a, -155	AD		[86,87]
miR-9, -124a, -125b, -128, -132, -219	AD		[62,67]
miR-494	PD	DJ-1	[110]
miR-7	PD	NLRP3	[113]
miR-205, let-7, -184*	PD	LRRK2	[40]
miR-1, -22*, -29	PD		[117]
miR-133b	PD		[105]
miR-132-5p, -132-3p, -143-5p, -143-3p, -574-5p	ALS	TDP-43	[135]
miR-206	ALS	HDAC4	[136]
miR-132	HD	P250GAP	[147]
miR-9, -9*	HD	REST, CoREST	[152]
miR-10b-5p	HD	BDNF	[159]
miR-34b	HD		[153]
miR-16-5p, -26a-5p, -29a-3p, -132-3p, -140-5p, -124a-3p, -146a-5p	Prion disease		[165]
miR-124a-3p, -136-5p, -376a-3p	Prion disease		[166]
miR-96	MSA	SLC6A6	[171]
miR-8	DRPLA		[173]

AD: Alzheimer’s Disease; PD: Parkinson’s Disease; ALS: Amyotrophic Lateral Sclerosis; HD: Huntington’s Disease; MS: Multiple Sclerosis; DRPLA: Dentatorubral-Pallidoluysian Atrophy
